# The microbial origin of marine autochthonous fluorescent dissolved organic matter

**DOI:** 10.3389/fmicb.2023.1152795

**Published:** 2023-04-12

**Authors:** Zhao Zhao

**Affiliations:** ^1^School of Marine Sciences, Sun Yat-sen University, Zhuhai, China; ^2^Southern Marine Science and Engineering Guangdong Laboratory (Zhuhai), Zhuhai, China

**Keywords:** marine microbes, FDOM's source, marine organic nitrogen, marine organic carbon, microbial pigments

Dissolved organic matter (DOM) in the ocean is a complexity with high diversity in chemical compositions. Diverse organic compounds are essential in global biogeochemical cycles composed of biogenic elements, mainly carbon, nitrogen, and sulfur (Carlson and Hansell, 2015). A certain fraction of DOM is light-absorbing, referred to as chromophoric DOM (CDOM). An important subset of CDOM is fluorescent, especially fluorescent DOM (FDOM) (Nelson and Siegel, 2013). CDOM absorbs UV–visible light with the typical absorption spectra in the blue and ultraviolet wavebands (200–400 nm and 400–800 m), and FDOM is in a generally limited window of excitation and emission wavelengths (240 nm−500 nm in excitation and 300–600 in emission) (Stedmon and Nelson, 2015). The optical properties of marine DOM were comparable and widely used in biogeochemistry, tracing the fate and source of DOM in the ocean (Coble, 2007). Wide distributions of marine FDOM were investigated across the global ocean (Yamashita and Tanoue, 2008; Nelson and Siegel, 2013). Based on studies from hydrography, high concentrations of CDOM/FDOM were normally investigated in riverine (Coble, 1996; Opsahl and Benner, 1997; Fellman et al., 2009) and soil samples (Guéguen and Cuss, 2011). Lignin is an essential and well-known terrestrial biomarker that also presents properties in absorbance and fluorescence (Hernes et al., 2009; Yamashita et al., 2015). However, the details of fluorescent signals of marine DOM were not able to mimic those from lignin or other terrestrial FDOM (Vecchio and Blough, 2004; Yamashita et al., 2010; Andrew et al., 2013), and the turnover rate of lignin and other terrestrial DOM could weakly support the standing stoke of marine FDOM (Opsahl and Benner, 1997; Hernes and Benner, 2003; Benner, 2004; Mannino et al., 2008; Yamashita et al., 2015). The debate about whether the origin of marine FDOM is autochthonous or allochthonous is ongoing (Drozdowska et al., 2015; Yamashita et al., 2015; Chen et al., 2016; Kwon et al., 2018). Sediment leaking would be supplementary to the allochthonous origin but limited to the wide distribution of marine FDOM (Skoog et al., 1996; Burdige et al., 2004; Yang et al., 2012; Chen et al., 2016). There must be another constant and generous autochthonous origin in the ocean. It is the black box generally called biogenetic derivation or, more precisely, the microbial origin, before our dissection on one of the key primary producers, picocyanobacteria (Zhao et al., 2017).

Microbes in the sea are diverse in terms of taxonomy and functional groups. Their activities are closely correlated with the fate of DOM (Tranvik, 1992; Jiao et al., 2010, 2011; Kujawinski, 2011). Cyanobacteria were unique among all these known autotrophic and heterotrophic microbes with phycobilin pigments (Chakdar and Pabbi, 2016; Saini et al., 2018). These tetrapyrrolic-based light-harvesting pigments were auto-fluorescent and in different types according to the peptides linked to the core tetrapyrrolic structure (Battersby, 2000; Stadnichuk et al., 2015). In the EEM analyses of DOM from picocyanobacterial cultures, the optical properties closely resembled typical oceanic FDOM found in the deep ocean. With a further comprehensive bulk analysis with the high-resolution mass spectrum and nuclear magnetism, the degradation products of phycobilin pigments were targeted to be the candidate that contributed to the fluorescent signal in picocyanobacterial-derived DOM. The dominant groups of unicellular picocyanobacteria, belonging to genera *Synechococcus* and *Prochlorococcus*, were widely distributed in the global ocean and contributed to up to 40% of primary production. Hence, picocyanobacteria were proved to be the very first certain contributor to marine FDOM (Zhao et al., 2017; Zheng et al., 2021). Even with a rough estimation based on the laboratory per cell production and total standing stokes of the picocyanobacterial populations, we could hardly define the proportion of picocyanobacterial-derived FDOM to the total oceanic FDOM without a global survey coupled with in-filed trace estimation.

Fluorescence is a specific optical property of compounds that is based on their particular chemical structure and composition. The optical properties are correlated to the molecular structures of organic compounds, with light absorption resulting in the loss of electron energy during transitions from the excited state to the ground state. Fluorophores are more specific than chromophores because fluorescence occurs only when the electron transitions from the lowest excited state. Structures such as aromatic and unsaturated bonds contribute to the majority of CDOM chromophores, but fluorescent signals are more unique and complex and are limited to certain compounds (Stedmon and Nelson, 2015). The diversity of microbial taxa and metabolic functions offers a broad range of selection opportunities for the production of organic compounds with fluorescent properties.

Picocyanobacteria could be essential contributors to marine FDOM (Xiao et al., 2021), but others are still lined up on the waiting list of potential candidates. Clues were gained from the initial study. Not all microbial species can produce fluorescent compounds without the cellular structure or metabolism function basics. *Synechococcus* released FDOM components when cells were lysed by either viral infection (Zhao et al., 2017, 2019) or environmental pressure (Zheng et al., 2021). The degradation products of the cell structure materials contributed directly to the FDOM signals (Lian et al., 2021). The photosynthetic pigment of *Prochlorococcus* was divinyl chlorophyll a and b (Chisholm et al., 1992), which was not the proved to be the origin of pyrrolic degradation products. Phycobilin genes were found in the *Prochlorococcus* genome, indicating the capacity of their production and further contribution (Steglich et al., 2005). Eukaryotic algae that contributed mainly to the DOM production in eutrophic zones would become the most competitive candidates. The absorbance of diatoms or dinoflagellate cultures was evaluated but no detailed fluorescent signals have been reported yet (Rochelle-Newall and Fisher, 2002; Burdige et al., 2004). Chlorophylls are a type of porphyrin compound that contains pyrrole rings, which are known to contribute to fluorescence (Zhao et al., 2017). However, chlorophylls are not readily water-soluble, so they are unlikely to directly contribute to FDOM in the ocean. The photosynthetic-related pigments in all kinds of microbes should be targeted first and foremost due to the chemical structure of fluorescent compounds (Kramer and Herndl, 2004). Candidates' range could be not only limited to the well-known photosynthetic algae groups but also photosynthetic prokaryotic microbes and other pigmented bacterial groups, such as the aerobic anoxygenic phototrophic bacteria (AAPB) (Yurkov and Beatty, 1998; Jiao et al., 2007; Ferrera et al., 2017) and proteorhodopsin in bacteria and archaea (Béjà et al., 2001; Frigaard et al., 2006; Gómez-Consarnau et al., 2007; DeLong and Béjà, 2010), and even in viruses (Yutin and Koonin, 2012).

Beyond the demonstration of the chemical nature, picocyanobacterial-derived FDOM was nitrogen-rich components in chemical compositions. The results led to a further discussion that FDOM could contribute importantly to both the organic carbon and nitrogen pools. The microbial origin of FDOM, e.g., picocyanobacteria, would be an essential link to coupling the nitrogen and carbon cycles in the ocean *via* DOM. The direction of the field was that modified estimations from laboratory incubation expanded to *in situ* quantification, filling the gaps in conceptual ameworks and models.

In conclusion, microbes were proven to be the key allochthonous origin of marine FDOM. On account of their high diversity, the verifications need to be more precise and focused. Combined application of multi-techniques is necessary for future attempts to further define the chemical nature of marine FDOM, in terms of the chemical composition and structure analyses of bulk DOM as well as the genome and transcriptome studies of microbial cell structure and metabolism.

## Author contributions

The author confirms being the sole contributor of this work and has approved it for publication.
